# Improving the identification of priority populations to increase hepatitis B testing rates, 2012

**DOI:** 10.1186/s12889-016-2716-7

**Published:** 2016-02-01

**Authors:** Caroline van Gemert, Julie Wang, Jody Simmons, Benjamin Cowie, Douglas Boyle, Mark Stoove, Chris Enright, Margaret Hellard

**Affiliations:** 1 Centre for Population Health, Burnet Institute, Melbourne, Australia; 2 Department of Epidemiology and Preventative Medicine, Monash University, Melbourne, Australia; 3 Cancer Council Victoria, Melbourne, Australia; 4 WHO Collaborating Centre for Viral Hepatitis, Doherty Institute, Melbourne, Australia; 5 Department of Medicine, University of Melbourne, Melbourne, Australia; 6 GRHANITE™ Health Informatics Unit, Health and Biomedical Informatics Centre, University of Melbourne, Melbourne, Australia

**Keywords:** Hepatitis B, Prevention & control, Asian continental ancestry group, Electronic health records

## Abstract

**Background:**

It is estimated that over 40 % of the 218,000 people with chronic hepatitis B (CHB) in Australia in 2011 are undiagnosed. A disproportionate number of those with undiagnosed infection were born in the Asia-Pacific region. Undiagnosed CHB can lead to ongoing transmission and late diagnosis limits opportunities to prevent progression to hepatocellular carcinoma (HCC) and cirrhosis.

Strategies are needed to increase testing for hepatitis B virus (HBV) (including culturally and linguistically diverse communities, Aboriginal and/or Torres Strait Islander (Indigenous) people and people who inject drugs). General practitioners (GPs) have a vital role in increasing HBV testing and the timely diagnosis CHB. This paper describes the impact of a GP-based screening intervention to improve CHB diagnosis among priority populations in Melbourne, Australia.

**Methods:**

A non-randomised, pre-post intervention study was conducted between 2012 and 2013 with three general practices in Melbourne, Australia. Using clinic electronic health records three priority populations known to be at increased CHB risk in Australia (1: Asian-born patients or patients of Asian ethnicity living in Australia; 2: Indigenous people; or 3): people with a history of injecting drugs were identified and their HBV status recorded. A random sample were then invited to attend their GP for HBV testing and/or vaccination. Baseline and follow-up electronic data collection identified patients that subsequently had a consultation and HBV screening test and/or vaccination.

**Results:**

From a total of 33,297 active patients, 2674 (8 %) were identified as a priority population at baseline; 2275 (85.1 %) of these patients had unknown HBV status from which 338 (14.0 %) were randomly sampled. One-fifth (*n* = 73, 21.6 %) of sampled patients subsequently had a GP consultation during the study period; only four people (5.5 %) were subsequently tested for HBV (CHB detected in *n* = 1) and none were vaccinated against HBV.

**Conclusion:**

CHB infection is an important long-term health issue in Australia and strategies to increase appropriate and timely testing are required. The study was effective at identifying whether Asian-born patients and patients of Asian had been tested or vaccinated for HBV; however the intervention was not effective at increasing HBV testing.

## Background

Despite being a leading cause of HCC globally, the asymptomatic nature of chronic hepatitis B (CHB) infection has resulted in a large number of undiagnosed cases [1–3]. The majority of CHB infections are acquired at birth or during early childhood with between 80 and 90 % of infants infected with hepatitis B virus (HBV) at birth developing CHB, compared to 30 % of infections among people aged ≤6 years and 5 % among adults [4, 5].

Current estimates suggest there are 218,000 people living with CHB in Australia; over half (56 %) born overseas and one-third (38 %) of this group were born in the Asia/Pacific region, a region with several countries with high and intermediate CHB prevalence [3, 6]. Recent estimates suggest that 44 % of people living with CHB in Australia are undiagnosed [3]. Only 5 % of people living with CHB are currently receiving antiviral therapy [7], representing a minority of the estimated 10–25 % who would benefit from treatment [8–11]. Undiagnosed CHB can lead to ongoing transmission, while late diagnosis limits opportunities to prevent progression to HCC or cirrhosis [4]. Further, HCC is the fastest increasing cause of cancer mortality in Australia, with the annual number of new cases increasing from 1.8 to 5.2 per 100,000 population between 1982 and 2007 [12]. It is imperative that those living with CHB are diagnosed in an timely manner as treatment significantly reduces the risk of HCC [13], and early detection of HCC is associated with improved 5-year survival rates [14].

Australia’s Second National HBV Strategy 2014–2017 (hereafter referred to as the Strategy) includes the target of increasing the proportion of people living with CHB that are diagnosed from 56 to 80 % by 2017 [8]. Priority populations identified in the Strategy include people from culturally and linguistically diverse backgrounds, particularly people born in Asia, the Pacific or Sub-Saharan African background, Indigenous people, and people who inject drugs (PWID). These groups have been identified as having reduced access to and use of health services, including preventative health programs and cancer screening [3, 15–20]. Key to achieving the CHB diagnosis target is the collection and recording of demographic data in health care settings to identify priority populations; however, recording of ethnicity and/or country of birth and Indigenous status in general practice settings is low in Australia [21, 22]. Therefore, strategies are needed to improve identification of priority populations in general practice and to increase HBV testing and appropriate follow-up care, including vaccination or treatment.

This paper presents results of a pilot study conducted with general practice clinics to identify people at increased risk of HBV in three priority populations (Asian-born patients or patients of Asian ethnicity, Indigenous people or PWID), and increase HBV testing and, where appropriate, HBV vaccination in these populations.

## Methods

### Study design

A non-randomised, pre-post pilot study was conducted between December 2012 and April 2013 in three general practices located in Melbourne, Australia. General practices were purposively selected in suburbs with a high proportion of Asian-born residents [23] and with a high case-load of Asian-born patients. The study included the implementation of a system to increase identification of people in high risk populations and a call back system to increase HBV testing and vaccination where appropriate. Stakeholders, including non-governmental organisations working in the areas of cancer prevention and hepatitis management, GPs and hepatology specialists were engaged during the design of the intervention.

### Site selection

The three clinics were located in suburbs with varying Index of Relative Socio-economic Disadvantage (IRSD) scores. The IRSD is an index of socio-economic status developed by the Australian Bureau of Statistics (ABS) based on the most recent Census (2011) which summarises a range of information about the economic and social conditions of people and households within a geographic area. The mean IRSD score is 1000; lower scores indicate relatively greater disadvantage in general whilst higher scores indicate relative lack of disadvantage generally. In 2011 the ISDR range across Australia was 588-1191 [24]. Clinic sizes ranged from small (~2500 active patients) to large (over 20,000 active patients). Two clinics did not charge private fees in addition to the fee billed through the national health insurance scheme (“Medicare”, see http://www.humanservices.gov.au/customer/subjects/medicare-services, Table 1).

**Table 1:** Description of participating general practice clinics

	Clinic 1	Clinic 2	Clinic 3
Number of doctors	5	2	5
Total number of active patients	8959	2424	21,914
Index of Relative Socio-economic Disadvantage score	1088	1060	908
Public or private billing	Medicare payment plus some private fee	Bulk billed Medicare payment only (no private fee)	Bulk billed Medicare payment only (no private fee)

### Identification of patients from priority population and sampling

The source population were active patients defined as those who attended participating clinics within the previous 24 months, aged 18 years and over, and were a member of one of the three following priority populations: 1) Asian-born patients or patients of Asian ethnicity living in Australia; 2) Indigenous people; or 3) people with a history of injecting drugs (herein referred to as people who inject drugs, PWID) were identified (see below for these methods).

Asian-born patients or patients of Asian ethnicity living in Australia were identified using a United States-developed electronic name-list predictive of people with Asian ethnicity [25]. The methods of developing the name list have been previously described [25, 26]. In short, a list of 20,000 names was developed in the United States from Social Security and Medicare administration records as a predictive method to identify ethnicity from the six major Asian ethnic groups in the United States, including Chinese (including those from Hong Kong and Taiwan), Japanese, Filipino, Korean (North and South), Indian and Vietnamese. The name list was applied by the GRHANITE^™^ technology [27] to determine the eligible Asian ethnicity population within each practice. The name list has been validated for use in screening patients for risk of CHB in the Australian state of Victoria [28] and has been used effectively in the United States to identify patients to receive electronic prompts to increase HBV tests [26]. GRHANITE^™^ technology interfaced with the clinic’s electronic health record (EHR) and applied the name list creating a flag for a match on first name, surname of both. This flag was extracted, in addition to other demographic and clinical details contained within the EHR.

Indigenous people included those that were coded as Aboriginal, Torres Strait Islander or both in a clinics EHR. PWID are not routinely identified in EHRs, and this population was identified through electronic review of clinical notes from the previous four years to identify records that suggested a history of injecting drug use by patients (for example, opioid substitution therapy). It was not possible to differentiate between current and former injecting drug use and therefore people with clinical notes indicating both current and former injecting drug use are included.

### Electronic identification of electronic health record data extraction software

De-identified data for the eligible study population were then automatically forwarded to the study secure data repository. The de-identified data were extracted at the individual patient level as per the study ethics permitted with the extraction happening in conjunction with an opt-out patient consent policy. A unique number mapping to the EHR record number was extracted to allow for re-identification of patients in the intervention arm by the practice without researchers having any access to person identifiers.

### Intervention

A random sample of those identified as being in a priority population were generated at each clinic for the intervention; the number of patients randomly sampled differed between clinics based on available resources at each clinic for conducting the intervention. Patients that: 1) matched the name-list for both first name and surname or identified as Indigenous or PWID (the priority populations); and 2) with no history of HBV testing within the previous four years or with a testing history that indicated HBV susceptibility (see Table 1 below), were sampled. Randomisation was based on the month of birth of patients in the source population; patients selected included those born in September, October and November in all clinics and in the third and largest clinic, the sample was further refined to include patients born in even-numbered years only to ensure a manageable sample size for clinic staff overseeing the intervention.

The intervention comprised of an invitation to each member of the sample to attend the clinic for HBV testing or vaccination where there was evidence of HBV susceptibility. Methods for patient contact differed between clinics and were selected and implemented by clinic administration staff in consultation with the project team. Clinics 1 and 2 mailed invitation letters to patients and Clinic 3 undertook a combination of mailed letters and telephone calls. The invitation letter was in English only however telephone calls were made by bilingual administrative staff. Non-respondents were not followed up (that is, the sample population received one invitation only) and the majority of invitations were sent between September and December 2012; in one clinic, the final invitations were sent on 11 April 2013. Approximately one-third of the invitations were delivered each month during the invitation period to ensure a manageable workload at clinics. The intervention period differed for patients according to the month of invitation, and ended on 30 April 2013 for all patients to allow sufficient time for potential consultation in response to the intervention (i.e., allowing a minimum of approximately three weeks and maximum of six months following the intervention to have a consultation).

### Electronic review of EHR and data collection

GRHANITE^™^ automated data extractions provided both the pre-intervention baseline and intervention period follow up study data. Four years of retrospective data were collected in November 2011 from all clinics; retrospective data allowed for review of previous HBV testing and identification of patients from priority populations. A second data extract was collected in April 2013 containing data from November 2011 until April 2013, allowing comparisons to be made in the pre-intervention and post-intervention periods.

Information on patients, consultations, requests and the corresponding results were collated. Demographic data collected included sex, age at consultation (derived from date of birth), Indigenous status, country of birth, flag for name match to list of Asian names (yes/no) and the patient’s unique ID number (to identify patients eligible for the intervention and monitor the interventions impact). HBV testing history data collected included if a test was requested (yes/no), the date of the consultation, the date the test was conducted (restricted to tests after 1/8/2007) and test result; pathology test reports were stored as fixed-layout flat document (i.e., PDF) and results were identified through text mining.

Diagnostic tests for HBV collected in this study include hepatitis B surface antigen (HBsAg), hepatitis B surface antibody (anti-HBs) and hepatitis B core antibody (anti-HBc). Case definitions used to determine HBV infection and immunity status are presented in Table 2.

**Table 2:** Case definitions to determine HBV infection and vaccination status

Infection status	
Unknown HBV status	No history of HBV testing
Chronic hepatitis B	HBsAg detected and/or HBV viral load detected
Immunity status	
CHB-negative and HBV susceptible (not vaccinated and no evidence of natural immunity)	HBsAg not detected and Anti-HBs not detected
CHB-negative and immune (vaccine-derived immunity or natural immunity)	HBsAg not detected and Anti-HBs detected
CHB-negative with insufficient information to determine immunity status	HBsAg not detected and Anti-HBs unknown

*HBsAg* hepatitis B surface antigen, *anti-HBs* hepatitis B surface antibody, *anti-HBc* hepatitis B core antibody

### Outcome measures

Outcome measures included in this analysis were: 1) the number of people identified as a priority population that were previously untested; 2) the number of people identified as a priority population that were previously untested and who subsequently had a HBV test; 3) the number of people identified as a priority population that were previously untested and who subsequently had a HBV test and whose HBV test results indicated CHB infection; and 4) the number of people identified as a priority population that were previously untested and who subsequently had a HBV test and whose HBV test results indicated susceptibility and were vaccinated.

### Ethical approval

The project received ethical approval from the Royal Australian College of General Practitioners (RACGP) National Research and Evaluation Ethics Committee and an opt-out consent mechanism was implemented at the practice level.

## Results

### Description of source population

A total of 33,297 active patients were identified across the three clinics’ patient management systems at baseline. A total of 2674 (8 %) of these patients were identified as a priority population for HBV testing and comprised the source population for the intervention. Nearly all of the source population were classified as Asian-born or of Asian ethnicity (*n* = 2654, 99.3 %). Eighteen (0.7 %) of the source population were people who identified as Indigenous and two (0.1 %) were PWID.

The majority of the source population (*n* = 2275, 85.1 %) had unknown HBV status and 399 (14.9 %) had known HBV status. Among the 399 patients with known HBV status, over half were HBV immune (*n* = 222, 62.2 %), 78 (21.8 %) were susceptible to HBV (i.e., CHB negative but no evidence of vaccination or natural immunity), and 57 (16.0 %) were CHB-negative with insufficient information to determine immunity status. Ten percent of the source population was found to have chronic infection (*n* = 42, 10.5 %) Table 3).

**Table 3:** Description of source population

	*n*	%
Description of active patients		
Total number of active patients	33,297	
Description of source population	2,674	8.0 %
Method of population identification		
Patients with Asian ethnicity	2,654	99.3 %
Indigenous people	18	0.7 %
People who inject drugs or with a history of injecting drugs	2	0.1 %
HBV infection and immunity status of source population		
Patients with unknown HBV status	2,274	85.1 %
Patients with known HBV status	399	14.9 %
Chronic HBV detected (% among patients with known HBV status, *n* = 399)	42	(10.5 %)
Chronic HBV not detected (% among patients with known HBV status, *n* = 399)	357	(89.5 %)
Susceptible to HBV (% among patients with no evidence of chronic HBV, *n* = 357)	78	(21.8 %)
Immune to HBV (% among patients with no evidence of chronic HBV, *n* = 357)	222	(62.2 %)
Unknown immunity status (% among patients with no evidence of chronic HBV, *n* = 357)	57	(16.0 %)
Eligible patients in source population (Patients with unknown HBV status and Patients with known HBV status that were susceptible to HBV or with unknown immunity status)	2,409	90.1 %

**Table 4:** Intervention description and results

	*n*	%
Number of patients sampled for intervention (% of source population)	338	(14.0 %)
Sep-11	93	27.5 %
Oct-11	131	38.8 %
Nov-11	114	33.7 %
Reason for intervention invitation		
HBV vaccination (% of patients sampled)	78	23.1 %
HBV testing (% of patients sampled)	260	76.9 %
Intervention impact		
Number of patients recalled that subsequently consulted a GP during the intervention period	73	21.6 %
Number of patients that had a consultation and tested for HBV (% of patients that subsequently had a GP consultation)	4	(5.5 %)
Chronic HBV detected (% of patients that subsequently had a GP consultation and were tested for HBV)	1	(25.0 %)
Chronic HBV not detected (% of patients that subsequently had a GP consultation and were tested for HBV)	3	(75.0 %)
Number of patients that had a consultation and were vaccinated against HBV (% of patients that subsequently had a GP consultation)	0	(0 %)

A total of 2409 (90.1 %) of patients from the source population were eligible for sampling for the second phase of the study (that is, patients with unknown HBV status and patients with known HBV status that were susceptible to HBV or with unknown immunity status).

### Intervention description and results

Three hundred and thirty-eight (14.0 %) of the eligible source population were randomly sampled for the intervention. Approximately one-third of the sample was recalled each month; 27.5 % in month 1, 38.8 % in month 2, 33.7 % in month 3. Of the 338 patients that were sent a recall letter or telephone call, one-fifth (*n* = 73, 21.6 %) subsequently had a GP consultation during the study follow up period. Among the 73 that subsequently consulted a GP in the follow up period, only 4 (5.5 %) were tested for HBV, one of whom had CHB detected. None of the other three patients commenced vaccination for HBV in the study period (Table 4).

## Discussion

CHB infection is a major long-term health issue in Australia due to the large number of Australians from culturally and linguistically diverse communities born in regions with moderate and high CHB prevalence, however diagnosis rates are unacceptably low. In this study, we found that electronic review of EHR database was an efficient and reasonably successful method to identify patients at increased risk of CHB and determine their HBV testing history. However, among those identified at increased risk and who were invited to the clinic for HBV testing or vaccination, there was a low rate of subsequent consultations and an even lower rate of HBV testing.

In this study, only 2.5 % of people who were identified as at-risk of CHB had country of birth recorded (data not presented in table), and therefore using the name-list greatly increased the number of at-risk patients identified than would have been identified if traditional identifiers were used. Several countries utilise similar electronic name-based ethnicity classification systems to supplement population health datasets when ethnicity data is not collected or incomplete. For example, Nam Pehchan and OnoMAP software have been used extensively in health research internationally and show utility in identifying people from Asian backgrounds [29–32]. No name-based ethnicity classification system is perfect however, and alternative strategies to improve the collection of information on cultural and/or ethnic background should be considered in general practice. For example, the recording of other cultural backgrounds of patients (including country of birth and language) is currently a discretionary data item in to the RACGP Standards for General Practice [33], however it may increase data completeness if this were revised to ensure it is a mandatory item. In addition, incentives to increase data completeness could be considered. In the United Kingdom, incentivisation of ethnicity (under the Quality and Outcomes Framework) improved completeness of ethnicity data for newly registered patients in general practices from 30 % to 80 % [34].

Whilst it was efficient to extract routinely collected data such as Indigenous status, the use of these data also relies on high levels of data completeness. Measuring the real burden of disease in Indigenous people is complicated by under-identification of in administrative health data collections, including general practice [35, 36]. Recording Indigenous status is a mandatory data item for accreditation purposes in the RACGP Standards [33] however data completeness in general practice is low, with around 60 % of Indigenous people having their status recorded [21]. Data completeness in this study was lower (around 45 %) and very few Indigenous people were subsequently identified. The prevalence of CHB in Indigenous people is higher than non-Indigenous people, and is estimated to be around 4 % [37]. Therefore, considerable efforts are required to increase data completeness to an acceptable level in general practice in Australia to ensure those living with CHB are identified. The indigenous Māori ethnic group in New Zealand, who have an estimated HBsAg prevalence of 5.6 % [38], had similarly low levels of ethnicity data completeness in the early 2000s; the implementation of a population-based funding model adjusted for ethnicity led to higher completeness rates of ethnicity in general practice datasets [39, 40]. An incentivised program also exists in Australia - the Indigenous Health Incentive, operating through the Practice Incentives Program and implemented through Medicare Australia [41]. The incentives offered through the Indigenous Health Incentive differ from the New Zealand model in that practices receive individualised payments for chronic disease management rather a population approach based on a whole-of-practice profile that requires higher levels of data completeness; a population approach to incentivisation may improve the identification of Indigenous people in general practice datasets.

There is currently not an efficient method to identify PWID in EHRs and patient notes were the main source of information used to identify these patients. This has considerable ethical implications and has the potential to introduce bias to the classification of patients by priority grouping. Further examination of efficient and ethical approaches to identifying PWID in clinical datasets is warranted, however it should be noted mainstream general practices may not be the most appropriate place to identify PWID for HBV care models. Issues such as stigma and expertise and level of comfort of providers in mainstream services may hinder service delivery to PWID and integrated care models that comprehensively address the complex range of health and social welfare needs of PWID are more appropriate services to provide testing and vaccination [42–44].

Identifying people at increased risk is important and our intervention was successful at this. However if we are to reach the Strategy’s target of that 80 % of people living with CHB that are diagnosed by 2017 it is vital that those identified as being at increased risk are tested to confirm their HBV status and then receive appropriate follow up. For those at risk of HBV, appropriate follow up includes HBV vaccination whilst for those diagnosed with CHB it includes regular follow up. Our findings reveal an extremely low baseline testing rate, with approximately 10 % of patients identified as at increased risk of CHB having had a test for CHB at the participating clinic in the previous four years. A low testing rate is likely to have fed into a low diagnosis rate. Based on estimates of CHB prevalence among people born in Asia/Pacific (3.55 %) [3], at least 94 people attending the participating clinics are likely to be living with CHB yet only 41 cases of CHB were identified through the initial data extraction and one additional case was identified in this study. A proportion of those remaining undiagnosed pose ongoing risks in relation to HBV transmission and CHB related morbidity and mortality.

Our intervention of sending invitations to those identified as at risk failed to increase consultation rates above the average attendance for people in this age range [45], and failed to increase HBV testing and vaccination in priority patients who attended the clinic in the study follow up period. Among those that did have a consultation, around 5 % subsequently had a HBV test. The reason why a HBV test was not requested in the remaining 95 % of consultations is not known, however it is possible that GPs were not aware of the patients identified risk of CHB and that consultations addressed other health concerns. It should be noted that GPs were not prompted (electronically or otherwise) at this visit to offer HBV testing and therefore interventions that operate at a clinic administration-patient level, without consultation level prompting or involvement of GPs, may offer limited utility.

In addition to low levels of testing in those that returned to the clinics, feedback from practice managers who oversaw the intervention implementation emphasised the resource intensity of the manual invitation process (i.e., generating the recall list and generating invitation letters or making telephone calls) suggesting such a system would not be sustainable even if testing had been higher. This highlights the need to investigate alternative efficient and sustainable models to prompt HBV testing among priority populations such as point-of-care electronic health reminders (that is, electronic reminders delivered during patient consultations) or reminders delivered prior to consultations. The use of electronic health reminders for preventive care in general practice has increased in recent years, including clinician reminders delivered before and after consultations (33). Systematic reviews of electronic health reminders have reported inconclusive results on their impact to increase preventative care measures [46, 47], however it is suggested that reminders delivered during consultations may be more successful compared to reminders delivered before consultations [47]. To date only one study has evaluated the impact of electronic health reminders to prompt HBV testing; a small randomised controlled trial in the United States determined the effectiveness of electronic reminders (emailed to clinicians 24 h before a patients’ scheduled appointment) to increase ordering of HBV tests among adults with Chinese and Vietnamese surnames (using the same name list used in this study). The trial was highly effective, with HBV tests being ordered for 41 % of consultations in the group that received an electronic reminder compared to 1 % of consultations in the control group [26].

Several limitations in this pilot intervention are noted. First, the study was a pilot and thus minimum sample sizes to observe the impact of the intervention were not calculated. Second, clinics were selected for their close contact with the target population, and therefore are not representative of broader general practice in Victoria. Third, the screening tool only comprised of names from six major Asian ethic groups and is not representative of ethnic or geographic areas with moderate and high CHB prevalence globally. In the Australian context, it would be greatly beneficial if names indicative of Pacific and African origin were also included. Fourth, the definition of active patients was broad and included patients with one consultation only at the participating clinic in the preceding four years before the intervention; these patients may have received the invitation but presented to a different clinic or not received the recall at all due to out of date personal information. Finally, the period of follow up after invitation to the study may have missed additional members of the target population who attended a clinic after the intervention follow up period.

## Conclusion

CHB is an increasingly important long-term health issue in Australia and many other countries. This intervention was effective at identifying Asian-born patients and patients of Asian ethnicity who may be at increased risk of CHB but was not effective in increasing HBV testing in this priority population. Further research is recommended to identify efficient and sustainable strategies to increase HBV testing in priority populations, such as the use of within-consultation electronic health reminders.

## Abbreviations

anti-HBc: hepatitis B core antibody
anti-HBs: hepatitis B surface antibody
CHB: chronic hepatitis B
HBsAg: hepatitis B surface antigen
HBV: hepatitis B virus
HCC: hepatocellular carcinoma
HER: electronic health record
IRSD: Index of Relative Socio-economic Disadvantage
PWID: people who inject drugs
RACGP: Royal Australian College of General Practitioners
